# Advocating for Older Adults in the Age of Social Media: Strategies to Achieve Peak Engagement on Twitter

**DOI:** 10.2196/49608

**Published:** 2024-05-01

**Authors:** Reuben Ng, Nicole Indran, Luyao Liu

**Affiliations:** 1 Lee Kuan Yew School of Public Policy, National University of Singapore, Singapore, Singapore; 2 Lloyd’s Register Foundation Institute for the Public Understanding of Risk, National University of Singapore, Singapore, Singapore

**Keywords:** age advocacy, social media engagement, older adults, ageism, data science

## Abstract

**Background:**

Over the last decade, many organizations dedicated to serving the needs and interests of older adults have turned to social media platforms, such as Twitter, subsequently rebranded X, to improve the visibility of age-related issues. However, notwithstanding their growing digital presence and participation, minimal attention has been paid to the use of social media among these advocacy groups. To achieve policy change, advocacy organizations must first be able to engage and mobilize audiences.

**Objective:**

Our study aims to elucidate how different tweet features affect the time it takes for posts uploaded by age advocacy organizations to reach peak engagement.

**Methods:**

We collated 204,905 tweets from 53 age advocacy organizations posted over a 12-year period. The engagement score of each tweet was calculated by combining well-established metrics, namely likes, retweets, quote tweets, and replies. We ran Cox models with tweet features as predictors and time-to-peak engagement as the outcome. “Peak engagement” (event) refers to engagement scores above the 75th percentile, and “time” refers to months taken to reach peak engagement per tweet.

**Results:**

Approximately 1 in 2 tweets (n=103,068, 50.3%) had either no hashtags or just 1 hashtag. Around two-thirds (n=131,220, 64%) of the tweets included a URL. Visual information was highly underused, with most tweets not including GIFs (n=204,202, 99.7%), videos (n=199,800, 97.5%), or photos (n=143,844, 70.2%). Roughly half (n=101,470, 49.5%) of the tweets contained mentions and 9.3% (n=19,009) of tweets were replies. Only 4.5% (n=9285) of tweets were quote tweets. Most tweets were uploaded in the afternoon (n=86,004, 42%) and on a weekday (n=180,499, 88.1%). As hypothesized, features associated with peak engagement were the inclusion of visual elements like photos, which increased peak engagement by 3 times (*P<*.001), and the use of 3 or more hashtags (*P<*.001). Quote tweets increased engagement by 3 times (*P<*.001), as compared to regular tweets, controlling for account-level covariates. Tweets from organizations with a higher tweet volume were 40% less likely to reach peak engagement (*P<*.001).

**Conclusions:**

Social media as a networked platform has the potential to reach users on a global scale and at an exponential speed. Having uncovered the features that are more likely to reach peak engagement on Twitter, our study serves as an invaluable resource for age advocacy organizations in their movement to create a more age-inclusive world.

## Introduction

Over the last decade, many organizations dedicated to serving the needs and interests of older adults have turned to social media platforms, such as Twitter, consequently rebranded as X, to improve the visibility of age-related issues. However, notwithstanding their growing digital presence and participation, minimal attention has been paid to the use of social media among these advocacy groups. To achieve policy change, advocacy organizations must first be able to engage and mobilize audiences. Our study elucidates how different tweet features affect the time it takes for posts uploaded by age advocacy organizations to reach peak engagement. We define “age advocacy” as the act of supporting or championing initiatives that address the needs of older adults.

The advent of social media has been profitable to advocacy groups for multiple reasons. First, although the visibility of a social movement was formerly determined by its ability to make headlines [1], social media has democratized the process of activism, allowing social actors to bypass the lack of attention received by a particular issue [2]. Second, the exchange of information via social media channels is not constrained by geographical barriers, thus enabling the rapid diffusion of information worldwide [1]. Third, social media platforms are a cost-effective means through which information can be transmitted and awareness of social issues heightened. Fourth, social media facilitates interaction between organizations and the public, thereby fostering sociopolitical discussion and participation [3].

When using social media, organizations typically set out to engage followers by uploading content that resonates with audiences [4] especially in view of the constant influx of information on the internet [5,6]. A well-engaged audience is essentially proof that a particular account has content which audiences find valuable and meaningful. Over the years, this concept of engagement has gained popularity across myriad disciplines, including marketing, psychology, communication, public relations, and organizational studies [3].

Twitter is a microblogging service home to over 300 million active users monthly [7]. Although originally viewed as an avenue for personal communication, the social media platform has since been used by academics, policy makers, and advocacy groups to access, share, and disseminate information [8]. Given the growing presence of age advocacy organizations on Twitter, this study looks at how different tweet features affect the time taken to reach peak engagement for posts uploaded by these organizations.

Both marketing experts and academics have conducted research on the features that promote user engagement on Twitter [9-15]. Although it is clear that adding photos and videos improves engagement [10,14], it remains a scholarly crux when the best time to post is [9,10,14,15], what the ideal number of hashtags to include is [11,12,14], and whether quote tweets drive engagement. There is, therefore, a need to ascertain which tweet features are linked to greater user engagement for content uploaded by age advocacy organizations specifically.

To date, only 1 study has explored the concept of engagement in relation to tweets uploaded by age advocacy organizations [16]. However, this study did not consider the time taken to reach peak engagement, which is important for several reasons. First, the time taken to hit peak engagement may be viewed by potential funders as a key performance indicator, which is a signal of the ability of an organization to retain the interest of its user base and consequently be eligible for further funding. Second, being able to reach peak engagement within a short period of time is vital if age advocacy organizations happen to be posting about time-sensitive issues.

From a conceptual angle, this study is significant in that it is one of the first to develop a framework that age advocacy organizations can use to optimize their social media posts for increased engagement. Existing studies have traced the origins of age advocacy in the United States [17] and have covered the need to advocate for older persons [18-24]. Research on web-based age advocacy, however, remains conspicuously absent, with most social media analyses in the gerontological field analyzing attitudes toward older persons [25-32]. From a practical angle, this study provides organizations with a road map to raise consciousness of age-related matters, which is especially pressing given the increasing proportion of older adults in populations worldwide [33]. By successfully engaging audiences, age advocacy organizations will be able to spur collective action and create policy change.

The tweet features examined in this study include the number of hashtags, URLs, and mentions present in the tweet; whether the tweet contains a GIF, photo, or video; whether the tweet is a “quote tweet”—a retweet with a comment added by the account—or a “reply”; the time of day the tweet was uploaded; and whether the tweet was uploaded on a weekday or the weekend.

We sought to test 4 hypotheses. First, in light of past findings that the inclusion of hashtags predicts the likelihood of a post to get retweeted [9,10,12], we hypothesized that tweets with more hashtags would be quicker to reach peak engagement (hypothesis 1). Second, in line with evidence that visual information is usually more stimulating than textual information [34,35], we hypothesized that tweets with GIFs, photos, or videos would be quicker to reach peak engagement than those without (hypothesis 2). Third, since followers of age advocacy organizations are likely to include scholars and policy makers who may value dialogue, input, or commentary [9,12,36,37], we hypothesized that quote tweets would be quicker to reach peak engagement (hypothesis 3). Finally, consistent with prior research, which finds higher tweet counts to be associated with negative engagement [9], we hypothesized that tweets uploaded by accounts with a higher tweet count would be slower to reach peak engagement (hypothesis 4).

## Methods

### Data Set

As few studies have looked at age advocacy organizations on Twitter, we first consolidated a list of organizations by referring to various sources [38-40]. Next, we checked whether these accounts had a presence on Twitter. To build a more comprehensive list of accounts, we looked through the list of followers of these accounts and identified other organizational accounts with large followings using a snowball sampling method. The organizations were eventually chosen based on the following inclusion criteria: (1) the organization was based in North America; (2) the organization was dedicated to serving the needs and interests of older persons specifically; and (3) the organization had at least 1000 followers. In total, there were 53 accounts (Multimedia Appendix 1).

We retrieved the data using the Twitter application programming interface (API) v2, which was accessed through Twitter’s Academic Research Product Track [41]. The v2 full-archive search allows for the programmatic access of public tweets from the complete archive dating back to the first tweet in March 2006, when the application was created. Relative to what was achievable with the standard v1.1 API, the v2 API grants users a higher monthly tweet cap and access to more precise filters [42].

Tweets collected (n=403,426) covered a period of 12 years, from July 17, 2009, to October 8, 2021, with the start date as the earliest date a particular tweet from any of the sampled accounts was uploaded and the end date a week after October 1, 2021, which was designated by the United Nations as the International Day of Older Persons [43]. “Retweets” (n=118,454) were excluded since they are not original content. Similarly, tweets with zero engagement (n=80,065) were excluded, as our focus was to observe the time taken to reach peak engagement. Finally, due to glitches with the API during the period of data collection—there were inaccuracies in the number of “likes” received by certain tweets—a few posts (n=2) were excluded. The final data set comprised 204,905 tweets.

### Tweet Features (Predictors and Covariates)

Similar to earlier work [9], we divided the tweet features into 2 categories: tweet-level (predictors) and account-level (covariates) features. The tweet-level features include the number of hashtags, URLs, and mentions present in the tweet; whether the tweet contains a GIF, photo or video; whether the tweet is a “quote tweet” or a “reply”; the time of day the tweet was uploaded; and the day—weekday or weekend—the tweet was uploaded. Following past literature [14], we divided the time of day based on CST into the following periods: morning (6 AM to 11:59 PM), afternoon (noon to 16:59 PM), evening (5 PM to 8:59 PM), and night (9 PM to 5:59 AM).

Account-level features, which served as covariates in our modeling, were consistent across all tweets belonging to a given account. These covariates included the number of followers, the number of accounts followed, the total number of tweets, and whether the account was “verified.” Except for the last variable, all skewed account-level variables were log transformed. Multimedia Appendix 2 contains a list of definitions of terms used on Twitter.

### Time-to-Peak Engagement (Outcome)

Following Twitter’s data dictionary [44], we used “likes” (ie, the number of times a particular tweet has been liked by other Twitter users), “retweets” (ie, the number of times a particular tweet has been retweeted), “quote tweets” (ie, the number of times a particular tweet has been quoted by other Twitter users) and “replies” (ie, the number of times a particular tweet has been replied to) as a proxy for user engagement. Our measurement of engagement aligns with that of previous studies [9,10,12-14]. To model the temporal aspects of engagement, we applied methods from survival analysis [45,46], which involved operationalizing engagement as a time-to-event variable. “Peak engagement” (event) refers to engagement scores above the 75th percentile, and “time” refers to months taken to reach peak engagement per tweet.

### Analytic Strategy

First, we performed Kaplan-Meier analyses to assess differences in engagement between categorical features—type of tweet and presence or absence of visual elements, such as photos, GIFs, videos, hashtags, URLs, and mentions. Respective curves were compared using the log-rank statistic. Second, we ran Cox regression models to identify the tweet features significantly associated with time-to-peak engagement, controlling for account-level variables. Since tweets from the same account contained identical account-level information, the independent assumption did not hold. To achieve a more robust variance, we set different user IDs as clusters [47]. Model 1 consisted of tweet-level features. Model 2 contained tweet-level features, controlling for account-level variables as covariates.

### Ethical Considerations

Ethical approval was not deemed necessary, as all the data used were publicly available and anonymized.

## Results

### Descriptive Statistics

Approximately 1 in 2 tweets (n=103,068, 50.3%) had either no hashtags or just 1 hashtag. Around two-thirds (n=131,220, 64%) of the tweets included a URL. Visual information was highly underused, with most tweets not including GIFs (n=204,202, 99.7%), videos (n=199,800, 97.5%), or photos (n=143,844, 70.2%). Roughly half (n=101,470, 49.5%) of the tweets contained mentions, and 9.3% (n=19,009) of the tweets were replies. Only 4.5% (n=9285) of the tweets were quote tweets. Most tweets were uploaded in the afternoon (n=86,004, 42%) and on a weekday (n=180,499, 88.1%). Table 1 summarizes the descriptive statistics. With regard to engagement, the lowest score was 1, and the highest score was 18,558. The engagement score at the 75th percentile was 8. Of the 204,905 tweets, 48,103 received an engagement score above 8.

**Table 1.** Description of tweets (n=204,905) from 53 age advocacy organizations posted over 12 years.

Tweet-level variables		Values, n (%)^a^		*F* ^b^	*P* value^c^
**Number of hashtags**				859.3	<.001
	0 or 1	103,068 (50.3)			
	2	53,336 (26.0)			
	≥3	48,501 (23.7)			
**Number of URLs**				137.5	<.001
	0	61,346 (29.9)			
	1	131,220 (64.0)			
	≥2	12,339 (6.0)			
**Number of mentions**				1252	<.001
	0	103,435 (50.5)			
	1	65,869 (32.2)			
	≥2	35,601 (17.4)			
**GIF**				701.2	<.001
	No	204,202 (99.7)			
	Yes	703 (0.3)			
**Photo**				11,540	<.001
	No	143,844 (70.2)			
	Yes	61,061 (29.8)			
**Video**				894.2	<.001
	No	199,800 (97.5)			
	Yes	5105 (2.5)			
**Type of tweet**				4800	<.001
	Original tweet	176,611 (86.2)			
	Quote tweet	9285 (4.5)			
	Reply	19,009 (9.3)			
**Time of upload**				498.2	<.001
	Afternoon	86,004 (42)			
	Evening	28,606 (14.0)			
	Morning	81,041 (39.6)			
	Night	9254 (4.5)			
**Day of upload**				132.1	<.001
	Weekday	180,499 (88.1)			
	Weekend	24,406 (11.9)			

a Percentages may not add up to 100 due to rounding.

b *F* refers to the *F*-statistic for the ANOVA test.

c *P* values are for the ANOVA test.

### Kaplan-Meier Analysis: Differences in Engagement Across Tweet Features

**Figure 1.**
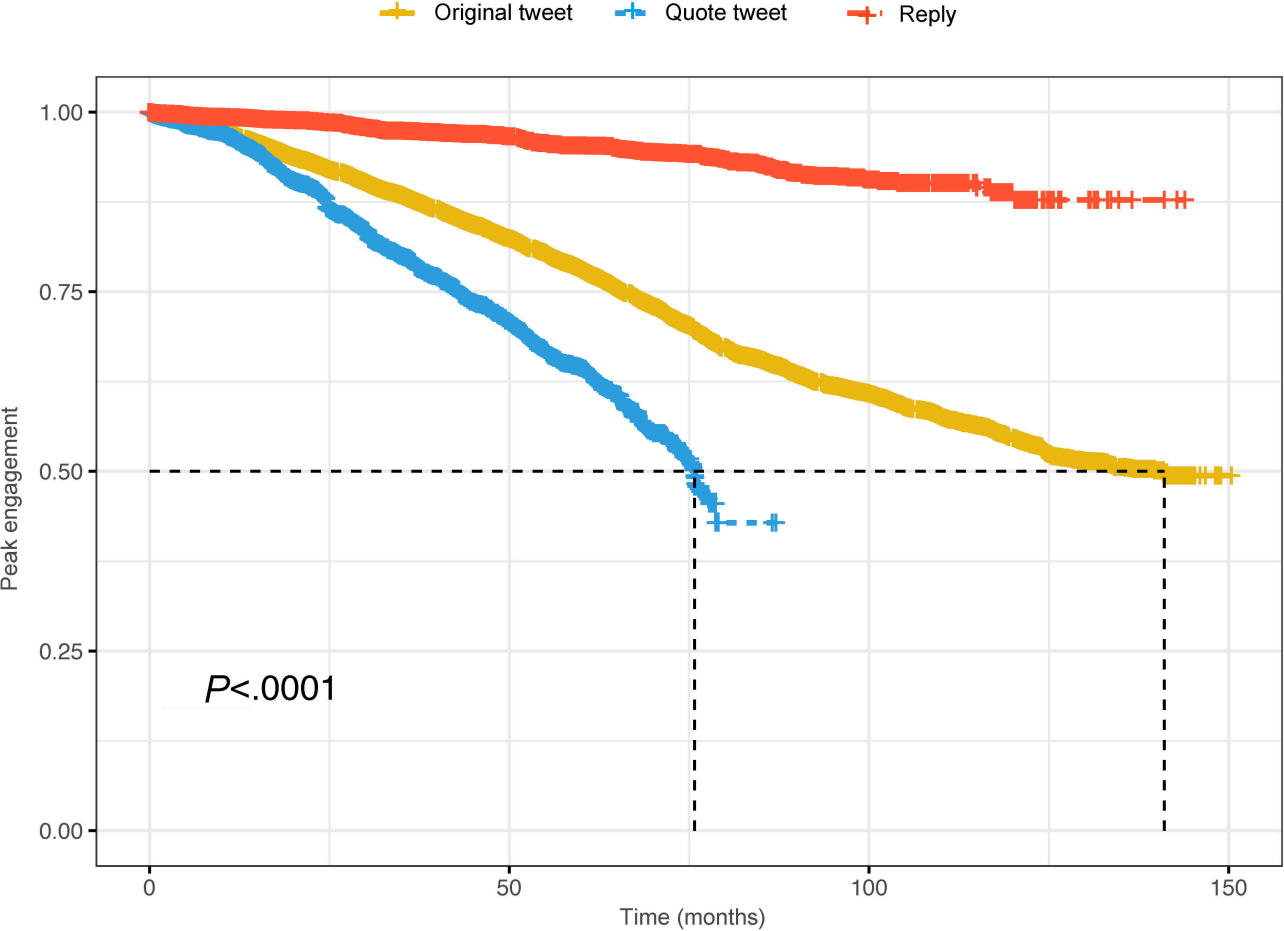
Engagement trajectories for 204,905 quote tweets, regular tweets, and replies posted over 12 years.

**Figure 2.**
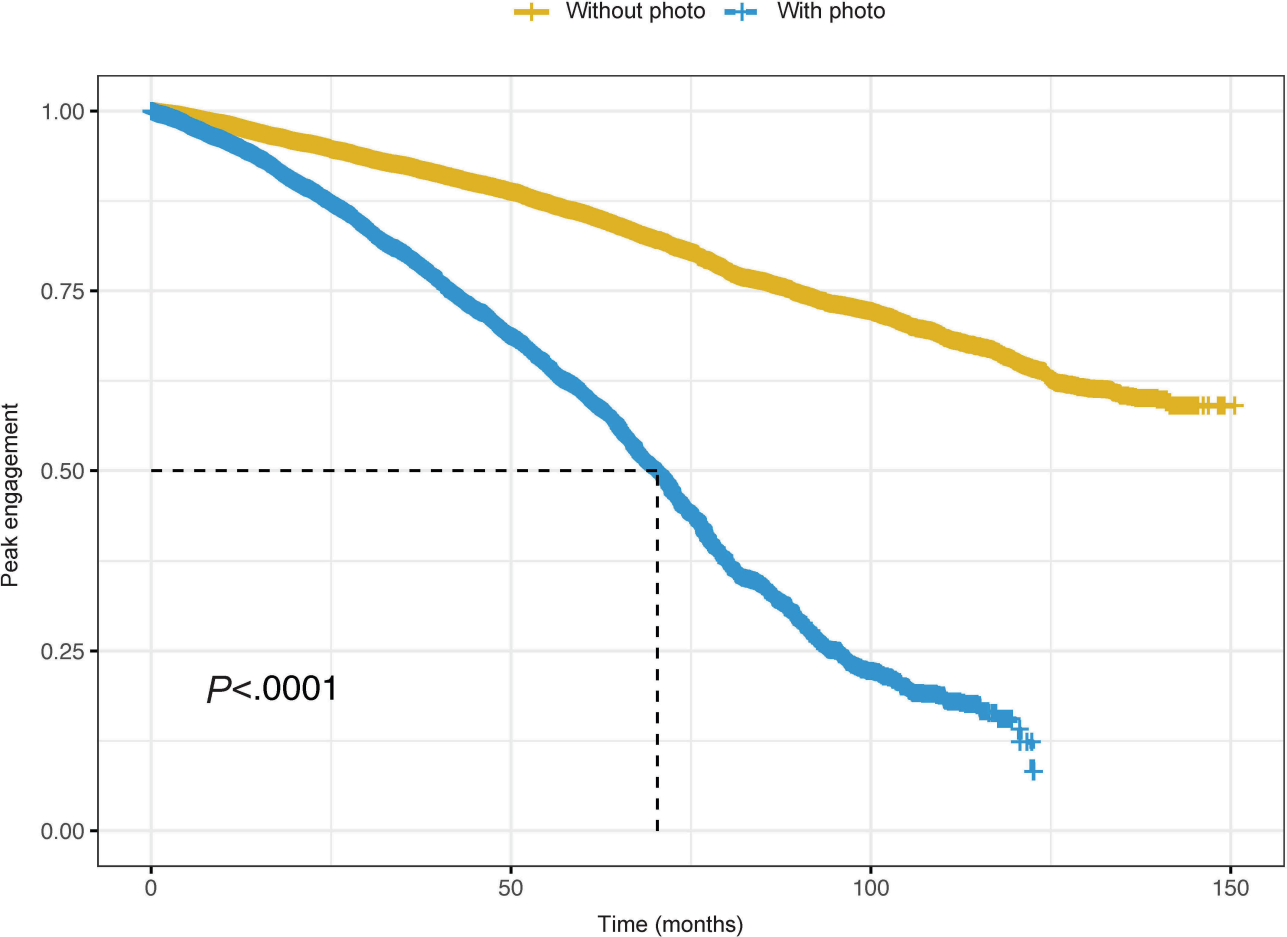
Engagement trajectories for 204,905 tweets with and without photos posted over 12 years.

We performed Kaplan-Meier analyses to examine differences in engagement across tweet features for 204,905 tweets posted over 146 months. Quote tweets achieved median engagement twice (log-rank test: *χ*^2^=3820; *P*<.0001) as fast as regular tweets (Figure 1). Specifically, there was an engagement advantage of 65 months, meaning that on average, quote tweets achieved peak engagement 65 months faster than regular tweets. Regarding visual elements, tweets with photos reached 75th percentile engagement 2.5 times faster than tweets without photos (log-rank test: *χ*^2^=1070; *P*<.0001), having an engagement advantage of 80 months (Figure 2). Similar results were observed for tweets containing GIFs (log-rank test: *χ*^2^=1070; *P<*.0001) and videos (log-rank test: *χ*^2^=8069; *P<*.0001) as compared to tweets without the respective features. Tweets with 3 or more hashtags had an engagement advantage of 14 months as compared to those with 2 hashtags (log-rank test: *χ*^2^=2700; *P<*.0001). Similar patterns emerged for URLs and mentions. Tweets with 2 or more URLs achieved an engagement advantage of 14 months compared to tweets with 1 URL (log-rank test: *χ*^2^=514; *P<*.0001). Conversely, tweets without mentions had greater engagement advantage than tweets with at least 1 mention (log-rank test: *χ*^2^=850; *P<*.0001).

### Multivariable Cox Regression: Tweet Features Associated With Time-to-Peak-Engagement

Tweets with 3 or more hashtags were 75% more likely to reach peak engagement than those with 1 or no hashtags (hazard ratio 1.75; *P<*.001), supporting hypothesis 1. Visual elements were particularly effective in nudging tweets toward peak engagement, being 4.25 times more effective for tweets with photos (*P<*.001), 6.38 times more effective for tweets with GIFs (*P<*.001), and 9.97 times more effective for tweets with videos (*P<*.001). This provided support for hypothesis 2. Consistent with hypothesis 3, quote tweets were 3.15 times more likely to achieve peak engagement (*P<*.001), as compared to regular tweets, controlling for account-level variables, such as the number of followers, the number of people followed, the number of tweets, and verified status. Meanwhile, at the account level, tweets posted by organizations with a higher tweet count were 40% less likely to reach peak engagement (hazard ratio 0.60; *P<*.001) as compared to those with a lower tweet count, providing support for hypothesis 4. The regression results are presented in Table 2. Coefficients reached significance at *P<*.05 after correcting for multiple comparisons using the Bonferroni method [48].

**Table 2.** Multivariable Cox models of tweet-level and account-level predictors of time-to-peak engagement for tweets (n=204,905) posted by age advocacy organizations over 12 years. Variables were log transformed.

Tweet-level variables		Model 1^a^		Model 2^a^	
		Hazard ratio(95% CI)	*P* value^b^	Hazard ratio(95% CI)	*P* value^b^
**Number of hashtags**					
	0 or 1	Reference	Reference	Reference	Reference
	2	1.19 (0.95-1.50)	>.99	1.19 (1.00-1.42)	.90
	≥3	1.50 (1.12-2.02)	.11	1.75 (1.37-2.22)	<.001
**Number of URLs**					
	0	Reference	Reference	Reference	Reference
	1	1.18 (0.94-1.48)	>.99	1.25 (1.07-1.45)	.07
	≥2	1.43 (1.01-2.01)	.62	1.44 (1.05-1.98)	.42
**Number of mentions**					
	0	Reference	Reference	Reference	Reference
	1	0.80 (0.67-0.97)	.32	0.84 (0.71-0.99)	.74
	≥2	0.95 (0.71-1.26)	>.99	1.03 (0.82-1.29)	>.99
GIF		8.63 (6.33-11.77)	<.001	6.38 (3.62-11.24)	<.001
Photo		4.06 (2.76-5.96)	<.001	4.25 (3.19-5.68)	<.001
Video		13.39 (6.06-29.58)	<.001	9.97 (3.36-29.65)	<.001
**Type of tweet**					
	Original	Reference	Reference	Reference	Reference
	Quote tweet	3.03 (1.96-4.68)	<.001	3.15 (2.02-4.90)	<.001
	Replies	0.41 (0.14-1.21)	>.99	0.28 (0.07-1.17)	>.99
**Time of upload**					
	Afternoon	Reference	Reference	Reference	Reference
	Evening	1.21 (1.01-1.45)	.63	1.09 (0.92-1.29)	>.99
	Morning	1.02 (0.90-1.15)	>.99	0.98 (0.86-1.12)	>.99
	Night	1.10 (0.81-1.48)	>.99	1.07 (0.73-1.57)	>.99
**Day of upload**					
	Weekday	Reference	Reference	Reference	Reference
	Weekend	1.00 (0.89-1.12)	>.99	0.98 (0.88-1.08)	>.99
**Account-level variables**					
	Follower count	—^c^	—	1.42 (1.17-1.73)	.009
	Friend count	—	—	1.60 (1.16-2.21)	.08
	Tweet count	—	—	0.60 (0.49-0.73)	<.001
	Verified status	—	—	1.20 (0.78-1.86)	>.99

a Constant not shown.

b *P* values have been adjusted using Bonferroni correction.

c Not applicable.

## Discussion

### Principal Findings

Although the technological era has ushered in numerous opportunities for advocacy organizations, scant attention has been devoted to examining the use of social media as a tool for age advocacy. As social media can be instrumental in fostering policy change, we sought to fill this gap by examining how different tweet features influence engagement for tweets uploaded by age advocacy organizations. Findings indicate that tweets that are more likely to reach peak engagement are those that include 3 or more hashtags, contain visual elements, or are quote tweets. In contrast, tweets posted by organizations with a higher tweet count are less likely to reach peak engagement as compared to those with a lower tweet count.

Unsurprisingly, tweets with hashtags are more likely to achieve peak engagement. Arguably the most iconic feature of Twitter, the hashtag is an organizational device that connects users to a broader community of individuals who use the same hashtag [1]. Although movements concerning race- or gender-related matters are notably associated with hashtags, such as #BlackLivesMatter and #MeToo, hashtags related to age have not garnered the same level of success. Nevertheless, several age advocacy groups have rolled out their own hashtag campaigns in recent years. For example, the American Association of Retired Persons started the hashtag #DisruptAging as a way to spark conversations on what it means to grow older [49]. Age Platform Europe, a network lobbying for the rights of older adults, began the #AgeingEqual campaign in 2018 to raise awareness of ageism [50]. More recently, the World Health Organization started the hashtag #AWorld4AllAges in a bid to encourage individuals to build a more age-inclusive world [51]. Moving forward, age advocacy organizations could consider embedding their tweets with more hashtags to improve the visibility of their content.

Peak engagement is also achieved when visual elements, such as GIFs, photos, or videos, are included in a tweet. That the brain absorbs and synthesizes visual information faster than textual information is an insight from past research [34,35]. Our results reveal that GIFs, photos, and videos are all piteously underused in content uploaded by age advocacy organizations. These organizations should therefore strive to include visual elements in their tweets to bolster their chances of capturing the attention of followers. Importantly, these elements should be carefully selected to avoid perpetuating visual ageism [52]. Organizations could consider selecting images from the newly launched Age-Positive Image Library, which houses images that portray old age more realistically [53].

Quote tweets reach peak engagement faster than original tweets. Whereas the retweet function enables users to repost a tweet verbatim, quote tweets give users the option of adding their own comments to the tweet being reposted and is often used by individuals who wish to express their opinions in the context of the original tweet [54]. Given how a large subset of those following age advocacy organizations likely comprises academics and policy makers—people who may rely on Twitter for sharing knowledge or participating in intellectual discussions [9,12]—it makes sense that quote tweets take less time to reach peak engagement. With less than 5% of the tweets collected being quote tweets, age advocacy organizations should consider using the quote tweet function more regularly to establish a dialogic relationship with the public.

As expected, having a high follower count lessens the time needed to reach peak engagement. Both older and newer accounts should therefore make concerted efforts to amass as many followers as possible. In particular, age advocacy organizations with little or no digital presence should prioritize crafting strategies to increase their follower count before attempting to bolster engagement.

Not spamming audiences with content is considered by marketing experts to be a basic rule of Twitter etiquette [55]. By posting too often, organizations risk losing public interest or frustrating followers [9]. In seeking to forge a connection with the public, organizations must exercise prudence with regard to how frequently they post to prevent inundating followers’ feeds. There are no hard and fast rules about how often to tweet, but social media managers of age advocacy organizations could monitor levels of engagement using the platform’s “Tweet Activity Dashboard” [55]. By tracking the level of engagement of each tweet, organizations will be able to gain insight into the optimal frequency for tweeting.

As age advocacy organizations curate their content with the goal of maximizing engagement, it is imperative that these organizations extend their outreach beyond researchers and policy makers to the larger society. This is especially critical since age-related issues have yet to gain widespread awareness among the public. Moreover, age advocacy organizations could involve older adults in the cocreation of initiatives, such as by collaborating with older influencers [56,57]. In addition, amid the prevalence of intergenerational tension in the digital sphere [29,30], there is a need to create opportunities for older and younger generations to interact. Hashtag campaigns could be used to encourage both generations to engage in meaningful dialogues.

### Limitations

This study has a number of limitations. First, the period that the tweets were posted is likely to have been a confounder in our analysis. It was only in 2014 that GIFs could be shared on Twitter. Likewise, the quote tweet feature was introduced only in 2015. However, tweets uploaded from 2009 onwards were included in our data set. The fact that there are now many more users on Twitter also means that posts that were uploaded before the platform was popular were less likely to be well engaged with. Second, considering that our objective was to look specifically at organizations, we could not offer insight into the level of engagement of tweets belonging to influential activists who champion the rights of older persons. Third, age advocacy organizations that are newer to Twitter were not included in the study since they did not fulfill the inclusion criterion of having at least 1000 followers at the time of analysis. Fourth, it is important to highlight that some tweets may have been uploaded solely for the purpose of informing or educating the public, rather than with the goal of engagement [9]. Finally, whether or not digital engagement actually inspires real-world action remains a moot point. Future analyses could adopt survey-based techniques [58,59] to understand activists’ perceptions of digital activism and how it compares to traditional offline activism.

Despite these limitations, our study contributes to the field of gerontology by developing some practical guidelines for improving age advocacy efforts on Twitter. With research on this topic still at the outset, directions for future research are plentiful. Foremost among them is the need to construct a theoretical framework outlining the concept of age advocacy. Subsequent research could also explore how levels of engagement vary across organizations specializing in areas like retirement, housing, or health care. Additionally, it would be worthwhile to dissect the profile of followers of age advocacy organizations. This could include an analysis of the distribution of followers based on characteristics such as age, gender, and occupation.

### Conclusions

Social media as a networked platform has the potential to reach users on a global scale and at an exponential speed. Having uncovered the features that are more likely to reach peak engagement on Twitter, our study serves as an invaluable resource for age advocacy organizations in their movement to create a more age-inclusive world.

## Supplementary material

10.2196/49608 Multimedia Appendix 1 List of age advocacy organizations.

10.2196/49608 Multimedia Appendix 2 Definitions of terms used on Twitter.

## Abbreviations

API: application programming interface
