# Supplementary material for: Advocating for Older Adults in the Age of Social Media: Strategies to Achieve Peak Engagement on Twitter
Source: JMIR Aging. 2024 May 1;7:e49608. doi: 10.2196/49608 (PMC11084120; doi:10.2196/49608)
Supplement: Multimedia Appendix 1 [file aging-v7-e49608-s001.docx]

**Multimedia Appendix 1: List of Age Advocacy Organizations.**

| **Twitter Handle** | **Organization** |
| --- | --- |
| @alzassociation | Alzheimer's Association |
| @AARP | AARP |
| @AARPadvocates | AARP Advocates |
| @ASAging | American Society on Aging |
| @AgeWave | Age Wave |
| @LeadingAge | LeadingAge |
| @NCOAging | National Council on the Aging |
| @AARPFoundation | AARP Foundation |
| @NextAvenue | Next Avenue |
| @AARPI | AARP International |
| @AARPresearch | AARP Research |
| @AmerGeriatrics | American Geriatrics Society |
| @geronsociety | Gerontological Society of America |
| @sageusa | SAGE: Advocacy & Services for LGBT Elders |
| @Aging20 | Aging 2.0 |
| @CreativityAging | National Center for Creative Aging |
| @AARPBlackCom | AARP Black Community |
| @johnahartford | John A. Hartford Foundation |
| @DiverseElders | Diverse Elders Coalition |
| @BoomingEncore | Booming Encore |
| @UN4Ageing | UN for Ageing |
| @GIAging | Grantmakers In Aging |
| @AoAgov | Administration on Aging |
| @changingaging | Changing Aging Org |
| @justiceinaging | Justice in Aging |
| @NCEAatUSC | National Center on Elder Abuse |
| @ElderCareTeam | Elder Care Workforce Alliance |
| @ActiveRetirees | Alliance for Retired Americans |
| @_McKnightsSL | McKnight's Senior Living |
| @ConsumerVoices | National Consumer Voice for Quality Long-term Care |
| @Oldpplarecool | Old People are Cool |
| @NHCOA | National Hispanic Council on Aging |
| @PensionRights | Pension Rights Center |
| @AARPLivable | AARP Livable Communities |
| @AgelessAlliance | Ageless Alliance |
| @WeAreAgeist | We are Ageist |
| @HealthInAging | Health in Aging Org |
| @love4ourelders | Love For Our Elders |
| @HelpAge_USA | HelpAge USA |
| @AARP_ND | AARP North Dakota |
| @LinkedSenior | Linked Senior |
| @DisruptAging | Disrupt Aging |
| @sgsmgr | Southern Gerontological Society |
| @AgefriendlyNYC | Center for Healthy Aging at NYAM |
| @ElderJustice | Elder Justice Coalition |
| @NYCElderabuse | NYC Elder Abuse Center |
| @AgeStrongBos | Age Strong |
| @JHGeriatrics | Johns Hopkins Geriatric Medicine & Gerontology |
| @AgeFriendlyOH | AgeFriendlyOH |
| @SecondWindDream | Second Wind Dreams |
| @OldSchool_Info | Old School.Info |
| @AgeInAmerica | Age in America |
| @NAPCA_aging | National Asian Pacific Center on Aging |
