# Supplementary material for: Advocating for Older Adults in the Age of Social Media: Strategies to Achieve Peak Engagement on Twitter
Source: JMIR Aging. 2024 May 1;7:e49608. doi: 10.2196/49608 (PMC11084120; doi:10.2196/49608)
Supplement: Multimedia Appendix 2 [file aging-v7-e49608-s002.docx]

**Multimedia Appendix 2: Definitions of Terms Used on Twitter.**

| **Type** | **Feature** | **Description** |
| --- | --- | --- |
| Tweet-level | Hashtag | The number of hashtags (a word or phrase preceded by the # symbol) a tweet contains. |
|  | URL | The number of URLs (web addresses) a tweet contains. |
|  | Mention | The number of times a user mentions another account's username (indicated by the ‘@’ symbol) in a tweet |
|  | GIF | Whether the tweet contains a GIF (moving image). |
|  | Photo | Whether the tweet contains a photo. |
|  | Video | Whether the tweet contains a video. |
|  | Quote | Whether the tweet is a quote tweet, which is a retweet with a comment added by the account. |
|  | Reply | Whether the tweet is a response to another user's tweet. |
|  | Time of Upload | The time of day (morning, afternoon, evening, night) that the tweet was uploaded. |
|  | Day of Upload | Whether the tweet was uploaded on a weekday or the weekend. |
| Account-level | Follower Count | The number of users following a particular account (log-transformed). |
|  | Friend Count | The number of users followed by a particular account (log-transformed). |
|  | Tweet Count | The number of tweets uploaded by a particular account since the time it was created (log-transformed). |
|  | Verified Status | Whether the account has been 'verified' by Twitter. |
